# Draft genome sequence of a carbapenemase-producing *Escherichia coli* strain MB2513103 bearing OXA-1207 from Qatar

**DOI:** 10.1128/mra.01108-25

**Published:** 2025-12-29

**Authors:** Mohammed Suleiman, Anju Sharma, Humna Shafaq, Thabisile Xaba, Clement K. M. Tsui, Andres Perez-Lopez

**Affiliations:** 1 Department of Pathology, Sidra Medicine 187187 , Doha, Qatar; 2 Department of Biomedical Sciences, Qatar University 61780, Doha, Qatar; 3 Infectious Disease Research Laboratory, National Center for Infectious Diseases, Singapore, Singapore; 4 Lee Kong Chian School of Medicine, Nanyang Technological University 54761, Singapore, Singapore; 5 Faculty of Medicine, University of British Columbia 98587 , Vancouver, BC, Canada; 6 Department of Pathology and Laboratory Medicine, Weill Cornell Medicine - Qatar 36579 , Doha, Qatar; Fluxus Inc., Sunnyvale, California, USA

**Keywords:** OXA-1207, *E. coli*, CPO

## Abstract

Multidrug-resistant *Escherichia coli* is considered an urgent public threat, causing sepsis and urinary tract infections. We report the draft genome of a carbapenemase-producing *Escherichia coli* strain, MB2513103, harboring a *bla*_OXA-1207_ gene isolated in Qatar. The genome statistics, along with the sequence type and resistance mechanisms, are predicted.

## ANNOUNCEMENT

Carbapenemase-producing *Escherichia coli* (CPEC) represents a critical threat in clinical and public health settings. This species can produce carbapenemase enzymes, which hydrolyze carbapenems, the last-resort treatment against multidrug-resistant gram-negative infections (1–3). It is crucial to monitor the circulation of CPEC because carbapenemase-encoding genes are often located on plasmids, facilitating horizontal gene transfer between bacterial species and accelerating dissemination within healthcare settings (2, 4, 5). Here, we report the genome of the CPEC strain isolated from a rectal swab in a child at Sidra’s Medicine hematology-oncology unit. Rectal swabs are inoculated onto CHROMagar mSuperCARBA plates (CHROMagar, France). Plates are incubated for 24 h under aerobic conditions at 35 ± 2°C. Suspicious colonies with dark pink/reddish color growing on the chromogenic plates were identified using the matrix-assisted laser-desorption ionization time-of-flight mass spectrometry (MALDI-TOF MS) (Bruker, Germany), and carbapenemase production was confirmed by NG-Test CARBA 5 (NG Biotech, France). Antimicrobial susceptibility testing was performed using the BD Phoenix M50 system (Becton Dickinson, USA), and minimum inhibitory concentrations (MICs) were interpreted according to the Clinical and Laboratory Standards Institute (CLSI) guidelines 2024 (6). Ethics approval for this study was obtained from the institutional review board at Sidra Medicine (1988373-5).

The isolate was maintained on a blood agar plate at 35°C. DNA was extracted from colonies using the EZ1 platform (QIAGEN, Germany). DNA libraries were constructed with a Nextera XT Kit (Illumina, USA) and sequenced on the Illumina MiSeq with 2×300 bp paired-end. Raw reads were quality assessed using FastQC (v0.12.1) (7), trimming & filtering of low-quality reads and adapters were done using Trimmomatic (v0.39) (8). Trimmed reads are assembled using SPAdes (v.4.2.0) (9) and quality is assessed with Quast (v5.0.2) (10).

Antimicrobial-resistant (AMR) genes and plasmids were predicted by AMRFinderPlus 3.11.20 (11) and MobSuite 3.1.9 (12) implemented in the SOLU bioinformatic platform with default parameters (https://www.solugenomics.com). The assembled genome was annotated by the NCBI Prokaryotic Genome Annotation Pipeline (PGAP) (accession number NZ JBRAXM010000000).

This isolate was identified as *E. coli* by MALDI-TOF. The OXA-48-like gene was detected by the Carba 5. This isolate exhibited low-level resistance to carbapenems and carried AMR genes such as *bla*_OXA-1207_, *qnrS1*, and *tet(B*) that confer resistance to multiple classes of antibiotics (Table 1).

**TABLE 1:** Summary of genome statistics and molecular mechanism of antibiotic resistance

Parameter	Data
BioProject ID	PRJNA1329010
Number of reads	821,250
Genome size (bp)	4,906,716
N_50_ (bp)	64,438
Number of contigs	199
Average coverage	50×
GC content (%)	50.67
Number of coding sequences	4,811
Number of coding genes	4,537
ST	617
Plasmid types predicted	IncFIA/IncFIB(pB171), Col(BS512)/Col(MG828), Col(pHAD28), IncX4, and IncFII
AMR genes and point mutations	bla_OXA-1207_, bla_TEM-190_, bla_CMY-145_, qnrS1, tet(B), _glpT E448K, nfsA G126R, gyrA D87N, blaTEM C32T, ftsI N337NYRIN, gyrA S83L, parC S80I, parE S458A_
Phenotypic antibiotic resistance profile	Ampicillin, Ampicillin-sulbactam, Aztreonam, Cefazolin, Cefepime, Cefoxitin, Ceftazidime, Ceftriaxone, Cefuroxime, Piperacillin Tazobactam, Ceftolozane Tazonactam, Ciprofloxacin, Levofloxacin, and Ertapenem.

The OXA-1207 is a newly reported, plasmid-encoded OXA-48-like carbapenemase, which was isolated from two *E. coli* isolates (ST405 and ST4405) in a German hospital (13). To the best of our knowledge, this is a new report for this *E. coli* ST617 strain carrying the OXA-1207 gene from the Middle East region. Insertional element ISKpn19 transposase and Tn3 family transposase were detected in the flanking regions of *bla*_OXA-1207_. A BLAST search of the contig (12,660 bp) bearing *bla*_OXA-1207_ was 99.99% to plasmids containing the OXA-48 family gene in various *E. coli* and *Klebsiella* isolates.

This multidrug-resistant isolate was sequenced as part of our ongoing surveillance in our hospital to prevent nosocomial outbreaks. This *E. coli* ST 617 has a single-locus variant within the ST10 complex and has been repeatedly reported from different regions with isolates originating from environmental and clinical samples (14–16).

## Data Availability

*Escherichia coli* strain MB2513103, whole-genome shotgun sequencing project has been deposited in NCBI under BioProject: PRJNA224116 (https://www.ncbi.nlm.nih.gov/bioproject/1329010), BioSample: SAMN51365783 (https://www.ncbi.nlm.nih.gov/biosample/SAMN51365783), SRA Study : SRP626145 (https://www.ncbi.nlm.nih.gov/sra/PRJNA1329010). Assembly: GCF 05772805.1 and NCBI Reference Sequence: NZ_JBRAXM000000000.1. This version of the project has the accession number NZ_JBRAXM010000000 and consists of sequences JBRAXM010000001-JBRAXM010000199.
